# 

**Published:** 1872-12

**Authors:** 


### INDEX OF SUBJECTS.

### ORIGINAL COMMUNICATIONS, ETC.

Resorption of a Fatty Tumor, .......... 713.

Chlorate of Potassa, .......... 716.

Selections—Nux Vomica in Dyspepsia, .......... 722.

Treatment of Spermatorrhœa, .......... 725.

Improved Pharmaceutical Formulæ, .......... 729.

A Remedy for Hæmoptysis .......... 731.

The Thermometer in Disease, .......... 732.

### EXTRACTS AND GLEANINGS.

Second Part.—Use of Nux Vomica in Certain Neuroses of Organic Life—Oxalate of Potash in Peritonitis, 739; A Positive Sign of Death—Resorts of Consumptives, 740; Injections of Ergot, etc.—Ozokerit in Skin Diseases, 741; Suppuration of one-half Lobe of Cerebrum—Differential Diagnosis, etc., 742; Effects of Bad Air Illustrated—Pill for Neuralgia—Anodyne Liniment in Otitis, 743; Neuralgia of the Testis—Turpentine Mixture—Bromo-Chloralum—Bromine as an Oxidizing Agent, 744; On the Employment of Creasote, etc., 745; Will Sulphur Prevent Conception—Ingrowing Toe-Nails—Arbor Vitæ in Small-Pox, 746; Soothing Syrups—The Latest Cure for Cholera, 747; A Simple Method of Arresting Epistaxis—Ammonia for Snake-Bites, 748; Alcoholic Treatment of Wounds, etc.,—The Castor-Oil Emulsion—Syphilis, etc., 749; Cider and Rheumatism—Gonorrhea—Healing of Ulcers by Serum, 750; Plugging the Rectum for Hemorrhage—Nitric Acid in Pertussis—Relation of Syphilis to Insanity, 751; Value of Myrrh—The Tincture of Eucalyptus as a Dressing for Wounds, 752; On the use of Ergot of Rye in Dysentery—A Good Nitro-Muriatic Bath—Sulphite of Soda in Variola, 753; Dogwood—Animalcules in Butter-milk—Oxide of Silver in Menorrhagia, 754; Warm Bath in Small-Pox—Anti Dyspeptic Pill in Cases of Atonic Dyspepsia—Railway Air-Cushion, 755; Nicotine in Tetanus—Sore Nipples—Chloride of Soda, 757; The Phosphate of Soda—Ointment for Itching of the Anus—Magnetic Wells, 758; Chloral in Asthmetic Bronchitis—For Cholera Infantum—Cerebro-Spinal Meningitis—Laxative Pill, 759; Inflammation of the Kidneys, etc.,—M. Barth, etc., 760; Cancer of the Tonsils, 761; Ointments for Glandular Swellings, 762; Fatal Case of Peritonitis, 763; Treatment of Asthma, 764; Syrups Cubebæ, 766; Toothache—Hint for Sea-Bathers, 766; Diagnostic Sign, etc.,—Dr. W. E. Whitehead, 767; Seneca in Bronchitis—Vermifuge, 768; Cause of Liver Complaint—Calumba in Vomiting, 769.

### EDITORIALS, REVIEWS, ETC.

Third Part.—To our Subscribers .......... 770.
The Close of the year, .......... 771.
Our Thanks—The Slaughter of the Innocents, .......... 772.
Communications Received—Advertising Department, .......... 775.
Obituary .......... 776.

